# What is equitable resilience?

**DOI:** 10.1016/j.worlddev.2018.04.020

**Published:** 2018-09

**Authors:** Nilufar Matin, John Forrester, Jonathan Ensor

**Affiliations:** Stockholm Environment Institute, Environment Department, University of York, YO10 5NG, United Kingdom

**Keywords:** Subjectivity, Inclusion, Cross-scale, Transformation, Social-ecological systems, Middle-range theory

## Abstract

Resilience has attracted criticism for its failure to address social vulnerability and to engage with issues of equity and power. Here, we ask: what is equitable resilience? Our focus is on what resilience does on the ground in relation to development, adaptation and disaster management, and on identifying critical issues for engaging with equity in resilience practice. Using techniques from systematic reviews, with variants of equitable resilience as our key search terms, we carried out an analytical literature review which reveals four interconnected themes: subjectivities, inclusion, cross-scale interactions, and transformation. Drawing on this analysis, we find that ‘equitable resilience’ is increasingly likely when resilience practice takes into account issues of social vulnerability and differential access to power, knowledge, and resources; it requires starting from people’s own perception of their position within their human-environmental system, and it accounts for their realities and for their need for a change of circumstance to avoid imbalances of power into the future. Our approach moves beyond debates that focus on the ontological disconnect between resilience and social theory, to provide a definition that can be used in practice alongside resilience indicators to drive ground level interventions towards equitable outcomes*.* Defined in this way, equitable resilience is able to support the development of social-ecological systems that are contextually rooted, responsive to change and socially just, and thus relevant to global sustainability challenges.

## Highlights

• The concept of resilience has often been critiqued as it underestimates issues of equity and power in human-environmental systems.
• This paper, based on an analytical literature review, reveals four themes essential in understanding equitable resilience in practice.
• The themes (subjectivities, inclusion, cross-scale interactions, and transformation) are embedded in a definition of ‘equitable resilience’.
• By proposing a middle-range theory, we expand the system to include social, cultural and political factors that distribute resilience outcomes.
• Equitable resilience can be applied alongside existing resilience indicators to drive resilience practice towards more equitable outcomes.

## 1 Introduction

Equity is concerned with how the *moral equality* of people can be realised. It places focus on the needs of those disadvantaged by relations of power and inequalities of opportunity, and how these barriers to human flourishing can be identified, understood and addressed (see for example, Rawls, 1971, Sen, 1999). From this perspective, the apparent failure of resilience to attend to the distributive and power dimensions of environmental and development problems is a serious limitation of the concept for analysis and practice. Authors such as MacKinnon and Derickson (2013) and Fainstein (2015) argue that resilience runs the risk of passivity, favouring the already advantaged and privileging existing social relations. Further, Folke et al., in a seminal paper setting out a social-ecological systems (SES) definition of resilience, recognise that, within the SES conceptualisation of resilience, “complex social dynamics, such as trust building and power relations, have often been underestimated and the view of social relationships simplified” (Folke’s, Hahn, Olsson, and Norberg, 2005, p. 462).

Folke’s et al. (2005) influential and widely cited definition states that resilience is the capacity of SES “to absorb disturbance and reorganize while undergoing change so as to still retain essentially the same function, structure, identity, and feedbacks” (Folke et al., 2005, p. 443). The limitations they recognise, arising from the treatment of the ‘social’ in resilience, have subsequently been noted from many perspectives. For example, in situations with goal and power conflicts (Jerneck and Olsson, 2008); when considering the nature of institutions as part of any resilience building initiative (Sjöstedt, 2015); or in designing processes of community participation around adaptation interventions (Bahadur and Tanner, 2014, Bahadur et al., 2013). For Hayward, the depoliticised language of resilience is not helpful in challenging “the drivers of social and economic change that threaten to destabilize our climate, increase social inequality, and degrade our environment” which require “rather less resilience and more vision for compassion and social justice, achieved through collective political action” (Hayward, 2013, p. 4).

For these reasons, while the practical application of resilience in international development and humanitarian contexts is a central concern for donors, policy makers and practitioners (Béné, Newsham, Davies, Ulrichs, and Godfrey-Wood, 2016; Elmqvist, 2017), questions surrounding the definition and operationalisation of resilience persist. While critical literature has done much to point out valid problems with both the meaning and the use of the word ‘resilience’, it has little to offer practitioners other than to point out that – from various disciplinary standpoints – resilience is a divisive rather than an integrating concept which needs to be “emancipated” from the natural sciences (Welsh, 2014, p. 21).

However, despite any apparent conflict between resilience and social theory, there is a burgeoning literature seeking to address social science critiques. Much of it is broadly consistent with the SES perspective offered in Folke et al.’s (2005) definition (see Ross and Berkes (2014) for one example). In 2012, Cote and Nightingale critiqued SES resilience– as it is practiced – using a “social theoretical lens”. According to them, although useful, the SES approach is found to be “inadequate in part because it repeats the weaknesses of earlier approaches in risk and hazard science that overemphasized the role of physical shocks and undertheorized that of political economic factors in conceptualizing vulnerability” (Cote and Nightingale, 2012, p. 478). Notwithstanding these caveats, they strongly support the role of the concept of resilience in bringing together academic disciplines to help understand the ‘messy’ nature of SES, and also helping to find a middle ground between science and practice.

Resilience researchers have sought to supplement current resilience thinking with other more socially grounded theories. For example, Adger, 2006, Walsh-Dilley et al., 2016 advocate for a rights-based approach; Brown and Westaway (2011) put forward human development and wellbeing approaches; Pelling and Manuel-Navarrete (2011) propose combining resilience with Giddens’ theory of power; Tschakert (2012) explores political ecology; and Tanner et al. (2015) find a livelihood perspective helpful in strengthening resilience thinking. Béné et al., 2014, Béné et al., 2016 suggest that a more ‘organic’ way to bring power and agency concerns more systematically into resilience thinking is to incorporate them directly into the conceptualization of resilience. In recognising the diversity of these contributions, Brown concludes that “a much greater engagement and reflection on social dimensions” (Brown, 2014, p. 114) has emerged within the resilience literature, while Weichselgartner and Kelman suggest that to overcome the sometimes narrow focus of resilience we need to foreground “the question of social transformation” (Weichselgartner and Kelman, 2014, p. 262). For Pelling, O’Brien and Matyas (2015), bringing transformation into resilience has the potential to disrupt inequitable development trajectories.

### 1.1 Equitable resilience

This paper makes a cross-disciplinary and analytical review of sufficient literature related to resilience to be able to contribute to the above debate and move past positions of polarisation, examining if and how resilience thinking in practice has addressed equity in the context of intersecting development, disaster risk management and climate change adaptation. In taking this approach, our aim is to develop a “middle-range theory” of equitable resilience (Geels, 2010). In common with Olsson, Jerneck, Thoren, Persson, and O’Byrne (2015), we advocate this approach in recognition that the “systems ontology” at the centre of resilience plays a role as a barrier, rather than as a bridge, to social science (see also Brand and Jax, 2007, Turner, 2010, Welsh, 2014). Likewise, the ontologies of social science ‘grand theories’ do not easily allow for integration and contextualisation, and often unravel in application (see for example Betz, 2016). Thus, rather than attempting to supplant, or transcend, one paradigmatic (‘grand’) theory with another, we find it more useful to accept that there are theories that have greater explanatory power at the grand-level, and theories that operate better at the “middle-range”, between “the all-inclusive systemic efforts to develop a unified theory” and “the minor but necessary working hypotheses that evolve in abundance during day to day research” (Merton, 1968: 39, quoted in Kang, 2014). Indeed, the defining point of middle-range theory is that it is empirically testable. By working towards theory at this level, we can better serve the interests of development and disaster risk policy and practice stakeholders, who engage with the world through the lens of particular problems in particular contexts (Kang, 2014). As Kallis and Norgaard (2010) point out, middle-range theory does not need to constantly refer back to grand-level, so it can operate independently of the argument and debate between grand-level theories (such as those between resilience theorists and their critics within the social sciences).

**Fig. 1:**
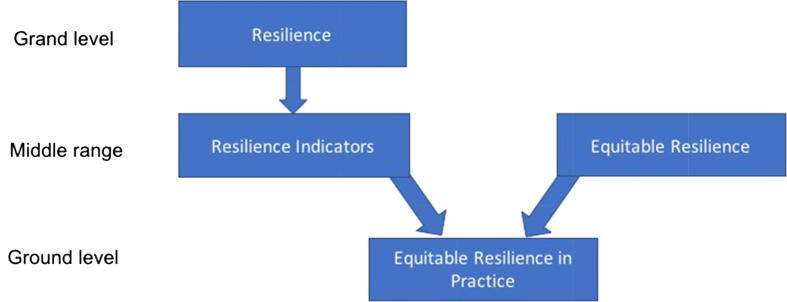
equitable resilience in practice – the application of equitable resilience in concert with resilience indicators or components.

**Fig. 2:**
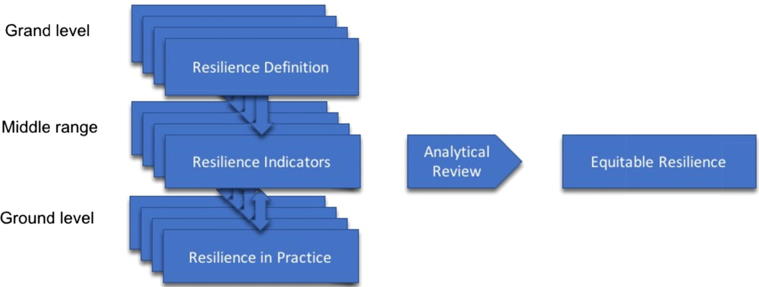
deriving equitable resilience from resilience literature that engages with equity in theory and practice.

Attempts to operationalise resilience in development and disaster risk management have for the most part focused on identifying critical components that can be acted on in practice (e.g. Béné et al., 2014, Plummer and Fennell, 2009, Berkes and Ross, 2013, Kruse et al., 2017). Bahadur et al. (2013), for example, offer ten resilience “characteristics” from literature focused on resilience in social, ecological and socio-ecological systems and applied to climate, disaster and development contexts. These indicators or components of resilience include ensuring multiple forms of diversity; securing effective governance and institutions; and addressing uncertainty and change. Our aim is to develop a definition of equitable resilience that can be used alongside resilience indicators such as these, in a given context, to drive ground level interventions towards equitable outcomes: we refer to this as *equitable resilience in practice* (Fig. 1). We recognise that there are different definitions or perspectives on resilience within the literature. Among them, we are focusing on those that address SES, in the context of development, risk, inequality and power within social systems. In keeping with our focus on the middle-range, we focus not on the concept of resilience *per se*, but on what it does on the ground in relation to our fields of focus (development, adaptation and disaster risk management and reduction). Equally, our intention is not to supplement one resilience theory with other socially grounded theories. Rather, we look to the literature to identify critical issues for engaging with equity in resilience practice. We aim to contribute to an understanding of what ‘equitable resilience’ means, in particular by bringing critiques of multiple conceptualisations of resilience together to find a common ground (Fig. 2). In so doing, we are drawing on resilience literature that has engaged with equity, to draw out insights and enable their systematic treatment in practice. Our analysis leads us to conclude that ‘equitable resilience’ can be defined as a form of human-environmental resilience which takes into account issues of social vulnerability and differentiated access to power, knowledge, and resources. It starts from people’s own perception of their position within their human-environmental system, and accounts for their realities, and of their need for a change of circumstance to avoid imbalances of power into the future.

### 1.2 Method

Our analytical review of the literature uses techniques informed by the cornerstones of systematic review: explicit and transparent literature sampling, selection, and approaches to analysis and synthesis (see, Petticrew and Roberts, 2006). We followed a four step process: first, determining research questions to guide the review; second, developing a search protocol (i.e., targeted databases and search terms) to explore literature databases; third, screening the results of the literature search based on their relevance to the research questions; and fourth, conducting analysis and synthesis of the remaining literature. We adapted the systematic review methodology in stage three (screening) to funnel-down through the thematic disposition of the remaining papers which helped drive our analysis forward in stage four. Further, in step four, our analysis was qualitative, which is to say that we did not code the texts.

To explore the equity implications of approaches to resilience, we framed the following two research questions. First, if and how current research on resilience in practice integrates (in)equitable social and power relations in conceptualising, describing and assessing the processes and outcomes of development pathways. Second, what are the essential features of resilience that must be built into a workable concept of equitable resilience that can inform practice. We limited these questions to the contexts of development, disaster risk and climate change adaptation in SES, and considered resilience in practice in terms of ground-level interventions, the resilience indicators or components that are used to operationalise resilience, and the overarching conceptualization of resilience that frames them (Fig. 2).

We chose the Web of Science (WoS) as the targeted database for our review. It contains a broad range of journals related to environmental management and governance, which are the principal topics of relevance to a grounded study of equity and resilience. The database was interrogated using keywords that comprised our search terms, identified collectively by the authors in a series of meetings, drawing on their knowledge and practical experience of the subject. These were resilience and: …equity, …equality, …power, …agency, …justice, …ethics, and …human rights. The analytical review was based on peer-reviewed journal articles published in the period 2005–2015 that appeared in the Social Sciences Citation Index (SSCI) of the Web of Science platform. The post-2004 cut-off date was selected to limit the data search but also to capture sufficient relevant literature that followed the Indian Ocean Tsunami. The identification, screening and eligibility assessment were thus done in accordance to the methodology used in the PRISMA (Preferred Reporting Items for Systematic Reviews and Meta-Analyses) flow diagram (see, www.prisma-statement.org/), as set out in Table 1.

**Table 1:** Analytical review steps.

Papers identified during Web of Science search (December 2015)	385
Papers identified from subsequent literature (up to March 2016)	80
Total papers, removing duplicates	400
Screening by authors for focus on social-ecological systems and engagement with equity and/or power	
Papers remaining for full text assessment	171

These 171 papers were then reviewed individually. From this review, four themes emerged that were deployed in the literature by researchers often as stand alone concepts, though at times in combination, to grasp equity and power issues in resilience. These provide the subsequent four sections below: subjectivities (Section 2); inclusion (Section 3); scale (Section 4); and transformation (Section 5). These four themes each form part of our definition of equitable resilience, and they arise from the significance of these themes within the reviewed literature to the achievement of equity. We provide an overall discussion of these themes in section 6 and offer conclusions in Section 7.

## 2 Equitable resilience and subjectivity

Subjectivity relates to one’s essential individuality – it is the lived experiences and affective states of individuals, patterned and felt in historically contingent settings, and mediated by institutional processes and cultural forms (Biehl, Good, & Kleinman, 2007). For Foucault, subjectivity contains two meanings: an individual is “subject to someone else by control and dependence; and tied to his own identity by a conscience or self-knowledge”. The consequences of both are felt in terms of power “which subjugates and makes individuals subject to” others (Foucault, 1982, p. 781). In current social and political discourse, subjectivity is considered both an empirical reality and an analytic category for assessing human nature, social control and agency. Thus, while subjectivities provide the object of study, the concept also provides the means to understand the values and institutions through which groups become socially differentiated, political identities are formed, and governance practices evolve.

Subjectivities are often grounded in individuals’ cultural, racial, ethnic, gender and other social attributes. Differential resilience results from the ability of individuals to mobilise these attributes in their favour. In other cases, where these attributes are socially constructed to discriminate against individuals and groups, they can subject them to further disenfranchisement, undermine their resilience, and create conditions for more risks to perpetuate. Subjectivities thus influence the processes that individuals, community and society employ to interpret hazards, their relationship with hazards, and the sources of information about hazards. Paton et al. suggest that people actively and constantly interpret stimuli from the environment, integrating these interpretations through a process of reflection with pre-existing mental models which incorporate their subjectivity and the “unique experiences people have accumulated during their lives” (Paton et al., 2010, p. 184).

There are a number of underlying processes and determinants of vulnerability and adaptation that arise from subjectivities of different forms. Socially produced contexts are one such phenomenon. Determinants of vulnerabilities can be linked to certain places and times (Tol and Yohe, 2007, Zou, 2012) and pre-disaster community contexts influence resilience after a disaster (Wickes, Zahnow, Taylor, & Piquero, 2015). Culturally derived values and beliefs surface as relevant and significant components of subjectivities that influence hazard mitigation (Paton et al., 2010, Turner et al., 2008) while, as Ribot notes, the differentiated causes of vulnerability in a given place need be traced “from that place through the social relations of production, exchange, domination, subordination, governance and subjectivity” (Ribot, 2014, p. 674).

A focus on the processes underpinning subjectivity allows one to explore the role of discourse and development processes in rendering individuals to forms of authority that can then be ascribed into policy or practice. Historical political and economic factors give rise to present day conditions, while contemporary events and processes directly and indirectly influence behaviours (Whaley and Weatherhead, 2014, p. 5). In this way, development processes may shift subjectivities and generate new social identities (Silva, Eriksen, & Ombe, 2010, p. 19). For example, the cases explored by Ratner et al. illustrate how “new resource claims by external actors disregard local institutions” or override significant social relationships that reach across ecosystems (Ratner et al., 2013a, p. 195). Similarly, in Mexico, Pelling and Manuel-Navarrete observe how the dominant discourse in development and disaster risk can promote the individualisation of wellbeing and risks. These narratives alter how people understand themselves in relation to others, forming new subjectivities that can undermine collective action and elevate personal goals. Their work found that most respondents saw development in personal terms (improvements in individual or family quality of life), potentially setting “a constraint for any transformational agenda and pos[ing] a challenge for adaptation and mitigation which might be seen as public goods” (Pelling and Manuel-Navarrete, 2011, p. 6). These cross-scale effects may set the stage for maladaptation (Barnett and O’Neil, 2010).

Subjectivities are also intersectional in the sense that social identities can cut across other attributes of individuals to produce and reproduce exclusion and discrimination (Evans, 2012, Evans, 2015, Kabeer, 2010, Nightingale, 2011). However, in other contexts, subjectivities can lead individuals to evade or resist particular processes that help (re)create them over time. Political identities can be formed where authorities divide people, explicitly or implicitly demarcating some as more powerful than others, and perpetuating or fostering unequal wellbeing and risks. This may challenge forms of subjection as well as open up possibilities for resistance that may either subvert or (when unsuccessful) entrench subjectivities (Nightingale, 2011, p. 161).

This literature highlights the significance of multiple subjectivities, how they shift over time, and how they connect to transformations in social systems. Drawing this out helps expose social power relations that have profound implications for generating or undermining resilience, as well as the persistence and distribution of resilience in different social groups.

## 3 Equitable resilience and inclusion

Overwhelming evidence argues for the inclusion in decision making of diverse social groupings that influence resource distribution and human-environmental relationships (including those based on gender relations, age, ethnicity, disability, sexuality, and other formal and informal groupings; e.g., see Connell and Messerschmidt, 2005, MacGregor, 2009, Tschakert, 2012). These characteristics reflect knowledge and risk perceptions indispensable for adaptation (Annear et al., 2014, Armas et al., 2015, Davies et al., 2014, Evans, 2012, Matarrita-Cascante and Trejos, 2013, Oven et al., 2012) and exclusion of certain groups from decision-making related to risk reduction and adaptation generally creates barriers to resilient transformation (Dominey-Howes et al., 2014, Evans, 2015, Wamsler and Brink, 2014). Tanner and Mitchell suggest that pro-poor adaptation can be “facilitated by improving our understanding of how age, gender, ethnicity, disability and other social factors constrain or enable adaptation opportunities and can potentially contribute to the realisation of climate justice and rights to adaptation” (Tanner and Mitchell, 2008, p. 3).

Integration of discourses and knowledges is often advocated for equitable resilience. Arguments are made for a more inclusive approach towards recognising different values and interests affecting adaptation outcomes, as well as their potential conflicts. In situations where adaptation responses taken by one group may affect the vulnerability context of other groups, or where strong vested interests within particular adaptation strategies may act as a barrier to sustainable adaptation, normative principles can be considered a first step towards social justice and environmental integrity (Eriksen et al., 2011). Ajibade and McBean argue for including a political ecology-inspired human rights discourse that can bring visibility to the hidden and socially constructed limitations faced by groups and communities (Ajibade and McBean, 2014, p. 76). Tanner et al. (2015) argue for linking aspects of human agency and rights to the livelihood approaches for wider transformational changes, while Ensor, Park, Hoddy, and Ratner (2015) integrate human rights principles into participatory research methods for analysing processes of marginalisation and exclusion in the aquatic agricultural systems in Timor-Leste. Arguments are made for legitimacy of cultural values and enfranchisement of indigenous knowledges in diverse contexts, such as among the First Nations communities in western North America (Turner et al., 2008); Aboriginal groups in Northern Australia (Hill et al., 2012, Howitt et al., 2012) and in Alaska (Cochran et al., 2013); and among communities at risk from tsunami in Indonesia (Seng, 2013). In discussions on flood risks and water governance in the UK, McEwen, Jones, and Robertson (2014) advocate inclusion for addressing power dynamics, while Whaley and Weatherhead, 2014, Whaley and Weatherhead, 2015 suggest a synthesis of political economy and local discourse analysis.

Addressing power asymmetries within and between formal and informal governance arrangements at different levels is major area of attention and concern (Aldunce, Beilin, Handmer, & Howden, 2016 see also our discussion of scale, below). Although power sharing is frequently viewed as a desirable outcome of these institutions, Whaley and Weatherhead suggest that power sharing this should also be embedded in process design, as “the balance of power between participants in the action situation intrinsically influences their behaviour and the sorts of outcomes that can be achieved” (Whaley and Weatherhead, 2014, p. 8). Barbedo et al. observe that as long as state institutions fail to promote coalitions between key stakeholders, these institutions are “prone to domination and strategic instrumentalisation” by stronger groups over the weak, contributing undesirable environmental outcomes and running “contrary to the very interests of each of the respective participants” (Barbedo, 2015, p. 9). Larsen et al. argue that “if resilience theory is increasingly proposed as the preferred approach by which disaster risk reduction is framed and implemented, it needs to acknowledge and incorporate much more explicitly this role of stakeholder agency and the processes through which legitimate visions of resilience are generated” (Larsen, Calgaro, & Thomalla, 2011, p. 489). For Wakjira, Fischer, and Pinard (2013) a key mechanism for adaptation is combining elements from both informal and formal institutions: they advocate inclusion of relevant elements of traditional institutions into new forms of governance as this can enhance their legitimacy and help future adaptation processes. Lebel, Wattana, and Talerngsri (2015) suggest building and creating ‘co-productive capacity’ in environmental governance that integrates scientific resources and governance capabilities in ways that bring about informed social change. Notwithstanding its importance, inclusive governance remains a challenge. A clear disappointment is evident in Whaley and Weatherhead’s comment on water resources management in England that, despite structural moves toward more participatory, cross-scale forms of water governance, government agencies “continue to exercise power over farmers and other nonstate actors instead of sharing power with them” (Lebel et al., 2015, p. 5).

## 4 Equitable resilience and scale

An appreciation of scale – geographical and temporal – is identified as central to both resilience and systems thinking about resilience. Vogel, Moser, Kasperson, and Dabelko (2007) argue that understanding scale-relevant roles (e.g. insider/outsider; stakeholder/knowledge provider) is paramount, yet note the relative paucity of inclusive methods to work across scales. The consideration of “multiple scales and temporal aspects [should result in a] greater understanding of global sustainability challenges” (Chelleri, Waters, Olazabal, & Minucci, 2015, p. 1) including societal equity as well as resilience. Further, scale plays a role in marginalisation, which may occur in relation to a geographic core, but can equally be socially or politically focused and as such needs to be recognised and understood as a function of multiple processes. Global organizations, including those concerned with economics (see Silva et al., 2010, passim), development (Perz et al., 2015, p. 12807) and disaster relief (Walker and Westley, 2011, p. 2) play “an increasingly visible and powerful role” (Olwig, 2012, p. 112) in development, further underlying the significance of scale to equity and resilience.

The potential for cross-scale effects of changes in resilience, and in particular how this intersects back into relations of power and marginality that determine available development pathways, is emphasised. Tschakert (2012, p. 2) draw attention to the significance of “multiscalar interactions, scalar dimensions of practice, and traversing scales” to understanding and addressing equity in resilience and development. Robards, Schoon, Meek, and Engle (2011, p. 522) argue for “greater attention to […] linkages across and among scales, and the idea that some ecosystem states at specific scales are more ‘desirable’ than others”. This acknowledgment of desirability brings in issues of subjectivity and inclusion. Oven et al. also note that “Vulnerability’ may be determined […] at different scales (individual, household, community, sub-national and national)” (Oven et al., 2012, p. 17).

Governance – both of the social system and the concomitant governance of the human-environmental system – is a critical scale-related aspect. Vervoort et al. note that in the “governance of social-ecological systems […] the role of scale has thus far largely been limited to the science arena”: they also note that issues of scale “are not just tools for the study of phenomena, but are deeply rooted in the structuring of actions from personal decisions to global policies” (Vervoort et al., 2012, p. 1). Bankoff argues that “effective leadership at the grassroots level” is vital to disaster risk management but that this power is often articulated through “alternative means” (Bankoff, 2015, p. 430) and thus vertical (cross-scale) collaboration becomes complex. Forrester et al. note that scale is “always influenced by competing perspectives and interests” and levels of governance as well as sectoral interests add to the complicatedness and well as complexity (Forrester, Cook, Bracken, Cinderby, & Donaldson, 2015, p. 202). While “collaborative governance” (Hill et al., 2015) can “accommodate multiple issues in decision making” (p. 276), Armitage et al. note that “further consideration of the role of power and marginality among groups participating” (Armitage, Marschke, & Plummer, 2008, p. 1) is needed, while Berardi et al. note that despite the emergence of tools aimed at integration, “[e]nvironmental governance initiatives at a range of scales … are rarely joined-up and are often undermined by other unsustainable initiatives put in place by the very same decision makers” (Berardi et al., 2015, pp. 2 & 13).

Multiple dimensions of scale may give rise to scalar conflict and unwanted cross-scale effects. These are made manifest in multiple forms. For example, where geographic communities exist at single scales, but communities of practice transcend scale, such as in local to national scale institutions and agencies (Begg et al., 2015, Chapin et al., 2015, Matin et al., 2015); where “coping and adaptive practices that work well at an individual or household level may be counterproductive at a larger scale” (Wamsler and Brink, 2014, p. 17); or when, for the poorest of the poor, to “be resilient, and for their communities to be resilient they need to be able to look beyond their immediate localities toward the response of the city and the state” (Walters, 2015, p. 55). Furthermore, too often locals are “pushed aside” by international forces and, as a result, international agencies incur local resentment (Scharffscher, 2011, pp. 71–72).

An important scalar conflict occurs where “costs are externalized”: this is evident from a temporal perspective in disaster relief where “although the specific resilience of the system to the immediate disaster may appear to have been addressed, the general resilience of the system may be decreased, making it more vulnerable to future shocks” (Walker and Westley, 2011, p. 4). Similarly, adaptation measures that are intended to improve resilience may simultaneously cause increased vulnerability at other scales. Put simply, “processes that increase resilience for some but not for others, and thereby increase inequity in society, cannot be considered sustainable.” (Beckman, 2011, p. 40). From a policy perspective, better understanding of “scalar limits to governance systems has the potential to benefit policy-makers concerned with how cross-scale risk governance might be facilitated in practice” (Blackburn, 2014, pp. 110–111).

## 5 Equitable resilience and transformation

The term *transformation* applies to situations where there are “nonlinear changes in systems or their host social and ecological environments” (Pelling et al., 2015, p. 113). The assumption that there is a system change means that transformation goes further than adaptation, which is more likely to be associated with incremental shifts in system performance (see. e.g., Plummer and Fennell, 2009, especially pp. 153–154). Indeed, transformation is invoked at the limits of adaptation “beyond which objectives and values can no longer be maintained through adaptation” (Preston, Dow, & Berkhout, 2013, p. 1012). Transformation includes both non-linear shifts in system functioning and also “the whole-scale breakdown of multiple institutions characterising a social system” (Davidson, 2010, p. 1145). It can be considered either as a revolution or as an extension of adaptation, but if the latter then it is one which “foregrounds questions of power and preference that have so far been underdeveloped in adaptation theory and practice” and, as such, thereby raises “distinct ethical and procedural questions for decision makers” (Pelling et al., 2015, p. 113).

While transformation suggests profound change, Wamsler and Brink note that it “might consist of a combined set of incremental improvements that transform coping systems from within” (Wamsler and Brink, 2014, p. 22). Ratner et al. similarly observe that should “changes in resource use patterns, accountability, and distribution of authority become sufficiently pronounced and lasting, it could be considered a transformation in the social-ecological system at this local scale” (Ratner et al., 2013b, p. 13). However, as Tanner and Mitchell discuss, adaptation processes that act to enhance poverty reduction rely “on institutional and governance structures that have both the incentives and ability to deliver services to support the needs of different groups and sectors” (Tanner and Mitchell, 2008, p. 3). Such institutional and governance reform may, in fact, need to be systemically transformational.

For many, transformation is inherently political and “responses must then be forged in the crucible of politics” (Ribot, 2014, p. 674). Similarly, Robards et al. (2011) “recognize the political nature of information required” to inform such responses (p. 523). If transformation means overcoming or rejecting dominant narratives that have persisted within a system, it also involves asking questions of who or what processes determine the object of resilience, and what contexts enable resilience winners and losers to emerge. For Pelling and Manuel-Navarrete, “[i]f we agree that the majority of contemporary social systems are unsustainable, then understanding how power is held and used is key to understanding how transformation is blocked or may be facilitated” (Pelling and Manuel-Navrrete, 2011, p. 2). The potential resides in transformation to open up new policies and practices, overturning established relationships of power and thereby to “address underlying failures of development […] by linking adaptation, mitigation, and sustainable development” (Pelling et al., 2015, p. 2).

Learning systems have a central role in enabling transformations. Social learning platforms, in which multiple stakeholders look to understand their different perspectives and forge new knowledge through joint learning and action, have the potential to foster and underpin “more democratic governance”, as stakeholders engage in processes of defining problems and solutions, “examining the drivers of change, and discovering differential vulnerability among actors” (Robards et al., 2011, p. 526). Engendering the capacity for such forms of learning “is key for transforming short-term disaster into longer term resilience” (Walker and Westley, 2011, p. 3). More broadly, these processes open spaces in which new understandings of environmental challenges and their settings may emerge. For Tanner et al. “Focusing on these transformational aspects of resilience helps us to consider radically different livelihood strategies that may be necessary to respond to climate change and the significant tradeoffs involved” (Tanner et al., 2015, p. 25). As Lof (2010) argues, “the resilience–learning–governance interface provides some fruitful insights for the conceptual and theoretical understanding of adaptability, adaptation and transformation in resilience theory” (Lof, 2010, p. 1).

The complexity and uncertainty associated with persistent challenges in environmental management have had profound implications for sustainability. While a shift to governance has “direct[ed] attention to broad participatory approaches”, at the same time, systems thinking has reframed sustainability “in terms of characteristics associated with resilience (e.g. capacity for self-organization, learning and change)”: yet such theory also “emphasises transformative changes and an integrative perspective that couples human and natural systems” (Plummer and Fennell, 2009, pp. 154 & 149). If the problem is systemic then solutions lie not in incremental adaptation, but in approaches that build towards systemic transformation. Thus, if equitable resilience means addressing underlying failures in development and disaster risk management, rather than perpetuating or sustaining them, it needs to open up possibilities for whole-scale transformation.

## 6 Discussion: Towards a middle-range theory of equitable resilience

Recent literature underlines the need for a 'middle-range' resilience theory that enables decision makers to engage with questions of equity. For example, Chelleri et al., 2015, Chelleri et al., 2016 demonstrate the need to address temporal and spatial scale to understand consequences of resilience, revealing the patterns of winners and losers inherent in scalar resilience “trade offs”. Resilience cannot be assumed to be the appropriate goal for policy in the same manner as sustainability (Elmqvist, 2017), and the search for sustainability may be better framed as a search for transformation, in particular in how governance operates to frame problems and potential solutions (Redman, 2014). The contribution of equitable resilience is to make clear the need to engage with such questions at the moment at which resilience is invoked in practice, enabling resilience to support the development of systems that are responsive to change and socially just, and thus relevant to global sustainability challenges (Chelleri et al., 2015).

Based on the analytical review of the literature set out above, we propose an operational and testable definition for equitable resilience:

Equitable resilience is that form of resilience which is increasingly likely when resilience practice takes into account issues of social vulnerability and differential access to power, knowledge, and resources; it requires starting from people’s own perception of their position within their human-environmental system, and it accounts for their realities and for their need for a change of circumstance to avoid imbalances of power into the future.

Our definition is embedded in the four important themes identified for equitable resilience: subjectivities, inclusion, scale, and transformation. Further, we recognise and highlight that there are significant interconnections and dependencies among these themes: subjectivities reveal how place, identity, and social contexts all come together to create a form of reality which influences the way people and communities see themselves and are treated by policy and the policy community. Likewise, genuine inclusion can be the *means* by which subjectivities can be addressed. Equitable resilience – in practice – needs to cross scale boundaries and allow for fundamental changes in the system in contexts where transformation is deemed desirable by the communities concerned.

Many of the reviewed papers have noted a form of interlinkedness among some of the four themes, but few explicitly address all four themes together. We argue, however, that all four need to be recognised as important if we are to engage with equity in resilience practice. A simplistic view that focuses exclusively on any one theme – or ignores their interlinkedness – may be insufficient. This is not to say that it will be necessary to give equal attention to each in every case, but an approach that seeks equitable resilience will need to account for all four. Equitable resilience is, therefore, inevitably context-specific. It is also a system outcome. For example, equitable resilience in a particular setting may demand a form of governance that embraces different types of communities and takes into account different levels of authorities, or integrates appreciation of subjectivities across the levels of governance to facilitate inclusion rather than as a way to exclude and deny people their rights. In these cases, attention to the interlinkages between the themes facilitates the inter-linking of context and system, forcing an expanded appreciation of the system in terms of the social, cultural and political relationships that distribute resilience outcomes.

Equitable resilience in practice, we suggest, thus requires contextualized investigation of the four themes through methods capable of revealing how actors and institutions (formal and informal) support narratives, practices or forms of regulation at different scales that subjugate or empower those whom ‘resilience in practice’ is intended to benefit. Resilience indicators alone are not enough to support this form of practice. For example, while Bahadur et al. (2013) go as far as explicitly including ‘issues of equity and justice’ among their ten resilience characteristics, in practice “it remains for practitioners to engage with critiques of resilience and acknowledge the potential for sustaining and reinforcing existing relations of power and resource access.” (Ensor, Park, Attwood, Kaminski, & Johnson, 2016, p. 14). Our analysis of the literature suggests that to “engage with critiques of resilience” requires systematic exploration of subjectivities, of the equity implications of inclusion and scale, and of the potential for transformation. The aim here is not to replace resilience interventions, but to complement them with ways of analysing for and engaging in resilience practice that, the literature suggests, increases the likelihood of equitable outcomes. While exploration of the research and practice methods to support this endeavour are not the subject of this study, the papers cited within our review offer numerous examples that attest to its feasibility.

## 7 Conclusion

This analysis has implications not only for conceptual and practical studies of resilience but also for wider attempts at human-environmental sustainability. The literature reviewed here supports our definition of *equitable resilience* as one which takes into account issues of power, subjection, and resistance; makes visible socially constructed limitations faced by groups and communities at all levels; and thinks about these issues in a joined-up way to avoid unsustainable interventions being made in the name of either disaster response or development.

As resilience becomes more prevalent in policy and practice, attention to the demands of equitable resilience becomes ever more pressing. Without expansion of resilience beyond policy discourses that focus on services, security and infrastructure, resilience practice will risk entrenching vulnerability and generating new risks for groups distributed across temporal and spatial scales. Put simply, this means allowing for a form of resilience which allows for systemic change, beyond adaptation. Operationalising equitable resilience will require policy and practitioner stakeholders to engage with the politics of social, cultural and political change. This may be felt as a significant new challenge, but it is one that is pressing and necessary.

Equitable resilience needs to be embedded in a system approach and go beyond consideration of equity in the processes and distribution of development outcomes, taking us much deeper into the complexity of social processes. Sharply defined notions of objectively identifiable ‘scientific’ resilience become much more blurred and messy in these middle-level social processes, and it is here that attention must be paid if equitable resilience is to result.

## 8 Conflict of interest

None.
